# Frank–Kasper Phases of Diblock Copolymer Melts: Self-Consistent Field Results of Two Commonly Used Models

**DOI:** 10.3390/polym16030372

**Published:** 2024-01-29

**Authors:** Juntong He, Qiang Wang

**Affiliations:** Department of Chemical and Biological Engineering, Colorado State University, 1370 Campus Delivery, Fort Collins, CO 80523, USA

**Keywords:** Frank–Kasper phases, diblock copolymers, conformational asymmetry, self-consistent field theory

## Abstract

We constructed phase diagrams of conformationally asymmetric diblock copolymer A-B melts using the polymer self-consistent field (SCF) calculations of both the dissipative particle dynamics chain (DPDC) model (i.e., compressible melts of discrete Gaussian chains with the DPD non-bonded potential) and the “standard” model (i.e., incompressible melts of continuous Gaussian chains with the Dirac *δ*-function non-bonded potential) in the *χN*-*ε* plane, where *χN* and *ε* characterize, respectively, the repulsion and conformational asymmetry between the A and B blocks, at the A-block volume fraction *f* = 0.2 and 0.3. Consistent with previous SCF calculations of the “standard” model, σ and A15 are the only stable Frank–Kasper (FK) phases among the five FK (i.e., σ, A15, C14, C15 and Z) phases considered. The stability of σ and A15 is due to their delicate balance between the energetic and entropic contributions to the Helmholtz free energy per chain of the system, which, within our parameter range, increases in the order of σ/A15, Z, and C14/C15. While in general the SCF phase diagrams of these two models are qualitatively consistent, A15 is not stable for the DPDC model at the copolymer chain length *N* = 10 and *f* = 0.3; any differences in the SCF phase diagrams are solely due to the differences between these two models.

## 1. Introduction

Recently, we proposed a model system (referred to as the dissipative particle dynamics chain (DPDC) model hereafter) that can be readily used in DPDs simulations of conformationally asymmetric diblock copolymer (DBC) A-B melts and performed the corresponding self-consistent field (SCF) calculations to study the stability of the Frank–Kasper (FK) phases formed by such A-B melts [1]. Our SCF phase diagrams of the DPDC model were constructed in the *χN*-*ε* plane, where *χN* and *ε ≡* (*b*_A_/*b*_B_)^2^ ≥ 1 characterize, respectively, the repulsion and conformational asymmetry between the A and B blocks, with *b*_P_ (P = A, B) being the effective bond length of the P block at the copolymer chain length *N* = 10 and the A-block volume fraction in the copolymer *f* = 0.2 and 0.3; among the five FK (i.e., σ, A15, C14, C15 and Z) phases considered, only σ was found to be stable [1]. Since A15 has been reported to be stable in previous SCF calculations [2,3,4] of the “standard” model (i.e., incompressible melts of continuous Gaussian chains, CGCs, with the Dirac *δ*-function non-bonded potential) [5,6] of A-B melts, to which our DPDC model reduces in a certain limit of *N→*∞ [1], here we construct an SCF phase diagram of the DPDC model at *N* = 20 and *f* = 0.3, where A15 is also found to be stable, and compare our phase diagrams of the DPDC model with those of the “standard” model also constructed in the *χN*-*ε* plane. Such comparisons highlight the effects of model differences on the SCF results of these two commonly used models. 

As pointed out in our previous work [1], while the “standard” model has been most commonly used in the study of block copolymer self-assembly due to its least number of model parameters, it simply cannot be used in molecular simulations and also causes ultraviolet divergence that is difficult to handle in field-theoretic simulations (FTSs) [7]. Given that the mean field approximation of the SCF theory neglects the system fluctuations and correlations, which are important to the low-molecular-weight DBC melts forming FK phases in experiments [3,8,9,10,11,12,13,14,15,16,17,18,19,20,21,22,23,24], the only way to unambiguously quantify its consequences is to directly compare the SCF results with those of molecular simulations or FTSs based on the same model system, thus without any parameter-fitting.

For the rest of this section, we give a brief overview of the relevant work in addition to those cited in our recent paper [1], again limiting our discussion to the spherical FK phases formed in neat A-B melts. The σ phase is reported in a new sample of poly(trifluoroethyl acrylate)-poly(dimethylsiloxane) by Ryu and co-workers [22], consistent with their previous work [9,19]; the formation of stable C14 in this system [9,19], however, cannot be explained by SCF calculations of neat A-B melts. Dong and co-workers synthesized a series of discrete (i.e., monodisperse) A-B melts with various block lengths, where the A block is an *oligo* lactide homopolymer and the B block is an *oligo* (*γ*-alkyl-*α*-hydroxyglutaric acid) homopolymer [23,24]; they achieved three *ε*-values (about 2.1, 3.4 and an “even larger” value) by increasing the side alkyl chain length in the B block [24] and found only σ at *ε* ≈ 2.1 [24] but both σ and A15 for larger *ε*-values [23,24]. 

Using SCF calculations of the “standard” model, Collanton and Dorfman performed geometric and thermodynamic analyses and found that a decrease in the A-B repulsion drives the phase transition from the body-centered cubic spheres to σ, leading to more polyhedral micelle cores, as well as that from σ to hexagonally packed cylinders as *f* further increases [25]. Fredrickson and co-workers reported SCF phase diagrams in the *ε*-*f* plane at *χN* = 60 for incompressible A-B melts of the “standard” model and of two discrete-chain (i.e., the discrete Gaussian chain and the freely jointed chain) models at *N* = 50 with the Dirac *δ*-function non-bonded potential [26] (we note that the SCF calculations of A-B melts using discrete-chain models with the Dirac *δ*-function non-bonded potential may lead to some unphysical results [27,28]); increasing *χN* in the “standard” model (compared to their previously reported SCF phase diagrams of this model [3]) expands the stable region in *f* of A15 into those of σ and cylinders, as well as the stable region in *f* of σ into that of body-centered cubic spheres, and replacing CGCs with discrete chains expands the stable region in *f* of A15 into that of σ [4]. The latter is also found for the SCF phase boundaries in *f* computed by Lequieu, who used a compressible model system similar to our DPDC model but with the Gaussian (instead of DPD) non-bonded potential, for *N* = 50 and 100 at *ε* = 9 and *χN* = 40, where the stable region in *f* of cylinders also expands into that of A15 [29]. 

Lequieu also proposed the “multi-representation” simulation (MRS) that combines particle-based (e.g., molecular dynamics, MD) simulation with field-based simulation (e.g., SCF calculation or FTS [30]) subject to the constraint that their spatial density profiles are the same; with his discrete-chain model at *ε* = 9 and *N* = 50, Lequieu demonstrated the usefulness of the MRS for (1) the accelerated equilibration of A15 at *f* = 0.3 and *χN* = 40 and (2) the study of single-chain dynamics in σ at *f* = 0.2 and *χN* = 40~80 both in MD simulations by mapping the SCF density profile to particle-based configurations, and for (3) the free-energy calculation along the evolution of a randomly initialized particle-based configuration to σ at *f* = 0.2 and *χN* = 35 in MD simulations by mapping each of the configurations taken from the MD trajectory to a field-based representation and computing the SCF free energy [29]. Note that a cut-off was used for the Gaussian non-bonded potential and that a particle-to-mesh approach was used to calculate the density profile in his MD simulation (which was needed to impose the constraint in the above mapping) [29]; the latter modified the non-bonded potential to be both position-dependent and anisotropic [31].

Finally, we note two recent reviews on FK phases formed by block copolymers [32] and by homogeneous soft matter [33] in general.

## 2. Models and Methods

### 2.1. The DPDC Model and Its SCF Calculations

Our DPDC model (referred to as the DPD model in our previous work [1,28]), which is a compressible system of discrete Gaussian chains with the DPD non-bonded potential, for conformationally asymmetric diblock copolymer A-B melts was explained in detail in our recent paper [1]. As also described there, we have extended the newly released C++/Cuda version [34] of PSCF [35], an open-source code for SCF calculations of the “standard” model for block copolymer self-assembly, to include our DPDC model. We therefore refer the readers to our recent work [1] for details.

### 2.2. The “Standard” Model and Its SCF Calculations

The “standard” model [5,6], which is an incompressible system of continuous Gaussian chains with the Dirac *δ*-function non-bonded potential, has been the most commonly used in SCF calculations of block copolymer self-assembly; we therefore refer the readers to Refs. [2,3,15] for its SCF calculations and just mention here that in the limit of the copolymer chain length *N→*∞, the effective bond length of the P(=A, B) block *b*_P_→0 (at finite $R\equiv \sqrt{N/6}b_{B}$, conformational asymmetry between A and B blocks *ε* ≡ (*b*_A_/*b*_B_)^2^ ≥ 1 and $b_{B}/r_{c}$ with *r_c_* denoting the range of the DPD potential) and the generalized Helfand compressibility parameter [36] *κ→*0, the DPDC model reduces to the “standard” model [1].

We have also improved the numerical performance of PSCF [35]. In particular, we have implemented the so-called Richardson extrapolated pseudo-spectral (REPS) methods for solving the one-end-integrated chain propagators from the modified diffusion equations (MDEs). With *R* taken as the unit of length, the forward propagator *q*(**r**,*s*), for example, satisfies the MDE $\frac{\partial q}{\partial s}=\varepsilon \nabla^{2}q-\omega_{A}(r)q$ for the normalized chain-contour variable 0 ≤ *s* ≤ *f* with the initial condition of *q*(**r**,*s* = 0) = 1, where *ω*_A_(**r**) is the conjugate field interacting with A segments at spatial position **r** and *f* the A-block volume fraction in the copolymer; the MDE has the formal solution of $q(r,s+ds)=\exp\left[\left(\varepsilon \nabla^{2}-\omega_{A}(r)\right)ds\right]q(r,s)$. Uniformly discretizing the chain contour into *n_s_* steps each of size Δ*s* = 1/*n_s_*, the 2nd-order pseudo-spectral (PS) method [37] gives $q(r,s+\Delta s)\approx \exp\left(-\omega_{A}(r)\Delta s/2\right)\exp\left(\Delta s\varepsilon \nabla^{2}\right)\exp\left(-\omega_{A}(r)\Delta s/2\right)q(r,s)$, which has a ***global*** error of *O*(Δ*s*^2^). Morse and co-workers first pointed out that the error of the PS method contains only even powers of Δ*s* and thus proposed a 4th-order method, which is used in PSCF [35], by linearly extrapolating the two results of *q*(**r**,*s* + Δ*s*) obtained via the PS method with the step sizes of Δ*s* and Δ*s*/2, respectively, to the limit of Δ*s→*0 [38]. This is similar to the (composite) trapezoidal rule for numerical integration, the error of which also contains only even powers of the step size. The *K*^th^-order polynomial extrapolation of the *K* + 1 results obtained via the trapezoidal rule with successively halved step sizes to the limit of zero step size then give the commonly used Romberg integration [39], with *K* = 1 corresponding to (composite) Simpson’s 1/3 rule. We, therefore, refer to the PS method and that proposed by Morse and co-workers [38] as the REPS-0 and REPS-1 method, respectively, and have implemented the REPS-*K* methods for *K* = 0, …, 4. Note that the REPS-*K* method has a global error of *O*(Δ*s*^2(*K*+1)^); this requires Romberg integration [39] of the same (or higher) order to calculate, for example, the volume-fraction field of A segments, $\phi_{A}(r)=\int_{0}^{f}ds\;q(r,s)q^{†}(r,s)/\hat{q}(q=0,s=1)$, where *q*^†^(r,*s*) denotes the backward propagator and $\hat{q}(q,s)\equiv \int dr\exp(-iq\cdot r)q(r,s)/V$ is the 3D Fourier transform of *q*(**r**,*s*) with **q** being the wavevector and *V* the system volume (for example, Simpson’s 1/3 rule is used in PSCF [35] to match the REPS-1 method), which in turn requires *n_s_f* (as well as *n_s_*(1 − *f*)) to be an integer multiple of 2*^K^*.

## 3. Results and Discussion

### 3.1. Unit-Cell Discretization and Accuracy of βf_c_

As explained in our recent paper [1], the SCF equations, including those for the minimization of the dimensionless (mean-field) Helmholtz free energy per chain *βf_c_*, where $\beta \equiv 1/k_{B}T$ with *k_B_* denoting the Boltzmann constant and *T* the thermodynamic temperature of the system, with respect to the (up to six) unit-cell parameters **θ**, are written as **f(x)** = **0** and solved via the Anderson mixing (AM) [40] to an accuracy of |**f**|_max_ < *ε*_0_ [41]. For the DPDC model, we set *ε*_0_ = 10^−10^ and choose the spatial discretization parameter *m* = 64 in our SCF calculations, which gives the accuracy of *βf_c_* for this model Δ(*m*) ≡ |*βf_c_*(*m*) − *βf_c_*(*m* = 128)| < 10^−8^ in all the cases at *χN* = 40 (and even smaller Δ for lower *χN*); see Figure 1 of Ref. [1] for details.

**Figure 1:**
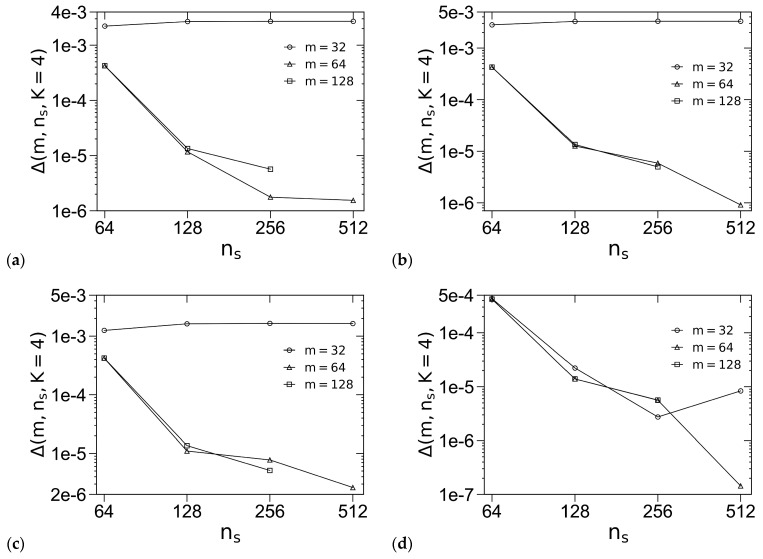

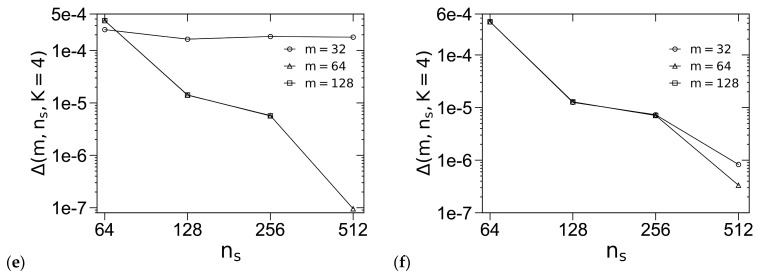
Logarithmic plot of the accuracy in the dimensionless (mean-field) Helmholtz free energies per chain *βf_c_* of (**a**) σ, (**b**) A15, (**c**) Z, (**d**) S_b_, (**e**) S_f_ and (**f**) C at *f* = 0.25, *χN* = 40 and *ε* = 4. The periodic unit cells of C, σ and all other phases (i.e., A15/Z/S_b_/S_f_) are uniformly discretized into *m*^2^, 2*m* × 2*m* × *m* and *m*^3^ grid points, respectively, and the chain contour is uniformly discretized into *n_s_* subintervals. The REPS-4 method is used to solve the MDEs. Note that the same unit-cell size, chosen to be very close to that in bulk (i.e., minimizing *βf_c_* of the corresponding phase), is used for each phase here. See the main text for more details.

For the “standard” model, we set *ε*_0_ = 10^−6^. Figure 1 shows how the accuracy of *βf_c_* for this model, denoted by Δ(*m*, *n_s_*, *K* = 4) ≡ |*βf_c_*(*m*, *n_s_*, *K* = 4) − *βf_c_*(*m* = 128, *n_s_* = 512, *K* = 4)|, varies with *m* and the chain-contour discretization parameter *n_s_* for various ordered phases, including regular-hexagonally packed cylinders (C), body-centered cubic spheres (S_b_), face-centered cubic spheres (S_f_) and three FK phases (i.e., σ, A15 and Z), where we use the REPS-4 method to solve the MDEs. We see that in most cases Δ decreases with increasing *n_s_* as expected; that Δ levels off with increasing *n_s_* at small *m* = 32 for σ, A15, Z and S_f_ indicates that the error caused by the real-space discretization dominates Δ in these cases. Furthermore, the Δ-curves for *m* = 64 and 128 nearly overlap in all the cases, indicating that *m* = 64 gives sufficient real-space discretization for all the ordered phases, the error of which is smaller than that caused by the chain-contour discretization (at the largest *χN*-value of 40 used in this work). We therefore also choose *m* = 64 in our subsequent SCF calculations of the “standard” model.

**Figure 2:**
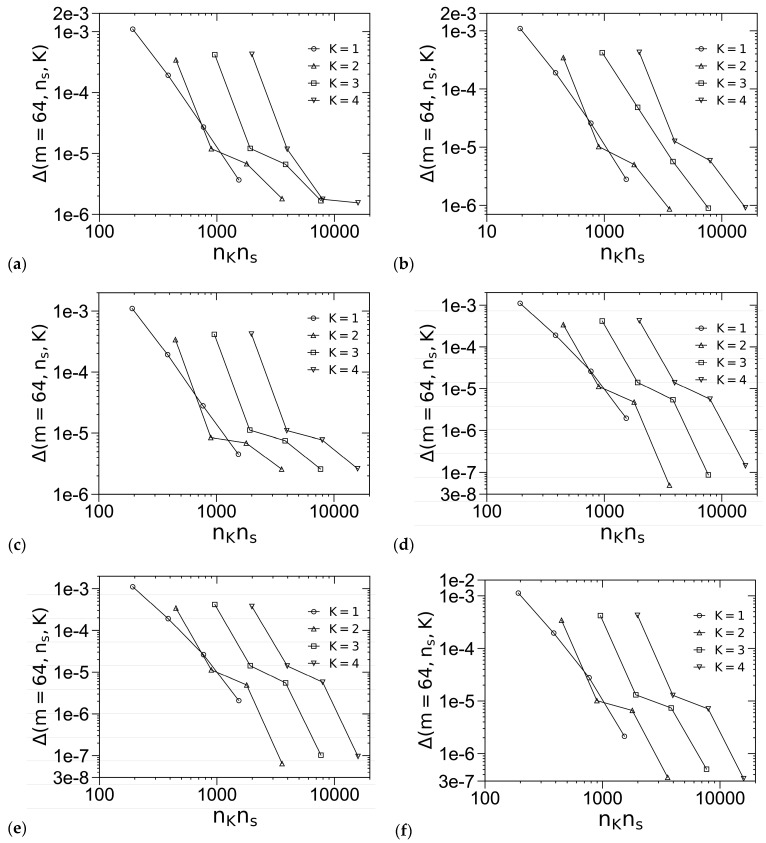
Logarithmic plot of the accuracy in *βf_c_* of (**a**) σ, (**b**) A15, (**c**) Z, (**d**) S_b_, (**e**) S_f_ and (**f**) C at *f* = 0.25, *χN* = 40 and *ε* = 4 for the “standard” model. The periodic unit cells of C, σ and all other phases (i.e., A15/Z/S_b_/S_f_) are uniformly discretized into *m*^2^, 2*m* × 2*m* × *m* and *m*^3^ grid points, respectively, and the chain contour is uniformly discretized into *n_s_* subintervals. The REPS-*K* method is used to solve the MDEs. Note that the same unit-cell size, chosen to be very close to that in bulk (i.e., minimizing *βf_c_* of the corresponding phase), is used for each phase here. See the main text for more details.

Figure 2 shows how Δ(*m* = 64, *n_s_*, *K*) ≡ |*βf_c_*(*m* = 64, *n_s_*, *K*) − *βf_c_*(*m* = 128, *n_s_* = 512, *K* = 4)| varies with *n_s_* and *K* for various ordered phases, where we use the REPS-*K* methods to solve the MDEs; note that, since the REPS-*K* method requires *n_K_* ≡ 2*^K^*^+1^ − 1 pairs of fast Fourier transforms to obtain the propagators in each step along the chain contour (with a step size of 1/*n_s_*), we use *n_K_n_s_* as the horizontal axis in Figure 2 in order to compare the efficiency of various REPS-*K* methods. We see that in all cases Δ decreases with increasing *n_s_* as expected, and that the REPS-*K* methods with *K* = 3 and 4 are less efficient than those with *K* = 1 and 2 (at least for the system parameters used in Figure 2), although the larger the *K*-value, the smaller the *n_s_*-value (thus the less the GPU memory) required to achieve a certain Δ. Based on Figure 2, we choose *K* = 2 with *n_s_* = 256 (at *f* = 0.25), which gives Δ < 10^−5^ (at the largest *χN*-value of 40 used in this work), in our SCF calculations of the “standard” model; in comparison, *n_s_* = 512 is needed for REPS-1 to reach this *βf_c_*-accuracy. Note that since each block must be uniformly discretized into an integer multiple of 2*^K^* subintervals in the REPS-*K* method, for *f* = 0.2 and 0.3 studied below, we use slightly larger *n_s_*-values of 260 and 280, respectively, in our subsequent SCF calculations.

Comparing Figure 1 of Ref. [1] with Figure 1 and Figure 2 here, we see that the SCF calculations of the DPDC model can achieve much higher *βf_c_*-accuracy than those of the “standard” model, due to the model differences. In particular, as pointed out in our recent paper [1], *N* in the DPDC model is much smaller than the *n_s_* (=100~1000) typically needed to achieve good *βf_c_*-accuracy for the “standard” model; SCF calculations of the DPDC model therefore are faster and use less memory, both by at least one order of magnitude, than the latter. In addition, *βf_c_* of an ordered phase in the DPDC model depends only on the spatial discretization, making its accuracy much easier to study than in the “standard” model.

### 3.2. Phase Diagrams

**Figure 3:**
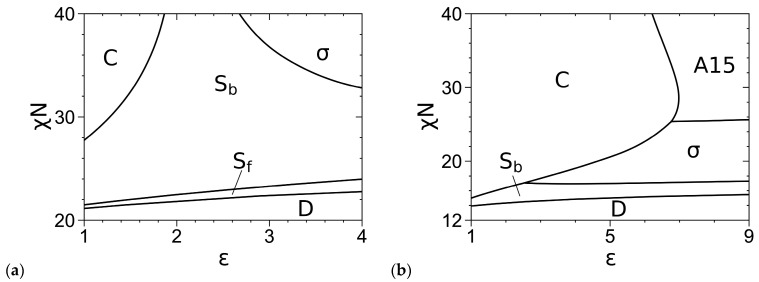
Phase diagrams of the “standard” model at (**a**) *f* = 0.2 and (**b**) *f* = 0.3, where “D” denotes the disordered phase. The periodic unit cells of C, σ and all other ordered phases (i.e., A15/Z/S_b_/S_f_) are uniformly discretized into 64^2^, 128 × 128 × 64 and 64^3^ grid points, respectively, and the chain contour is uniformly discretized into (**a**) 260 and (**b**) 280 subintervals. The REPS-2 method is used to solve the MDEs, and the phase boundaries are solved to an accuracy of 10^−5^ in *βf_c_*. The triple points are at (*ε*, *χN*) = (2.434, 16.944) and (6.752,25.321) in (**b**). See the main text for more details.

Figure 3 shows our SCF phase diagrams of the “standard” model in the *χN*-*ε* plane at *f* = 0.2 and 0.3, where the phase boundaries are solved via Ridders’ method [42] by equating, to an accuracy of 10^−5^, *βf_c_* of the two phases having the smallest *βf_c_* at given *ε* and the obtained *χN*. Table 1 quantitatively compares eight of our phase boundary points with those obtained by Fredrickson and co-workers [3]; note that their phase diagrams are in the *χN*-*f* plane at *ε* = 9 and in the *ε*-*f* plane at *χN* = 40. Our phase boundary points are in good agreement with theirs, with the largest relative deviation being about 2%. Since they considered but did not find C14 and C15 to be stable in their phase diagrams [3], we do not include these phases in our SCF calculations of the “standard” model. On the other hand, we have included but not found the Z phase to be stable. From Figure 3a, we see that at *f* = 0.2, increasing *χN* expands the stable region in *ε* of σ into that of S_b_, consistent with the phase diagrams for the “standard” model at *χN* = 40 [3] and 60 [4] obtained by Fredrickson and co-workers. From Figure 3b, we see that at *f* = 0.3, increasing *χN* < 28.580 expands the stable region in *ε* of C into those of σ and A15, but increasing *χN* > 28.580 expands the stable region in *ε* of A15 into that of C; the latter is also consistent with the phase diagrams at *χN* = 40 [3] and 60 [4] obtained by Fredrickson and co-workers.

**Figure 4:**
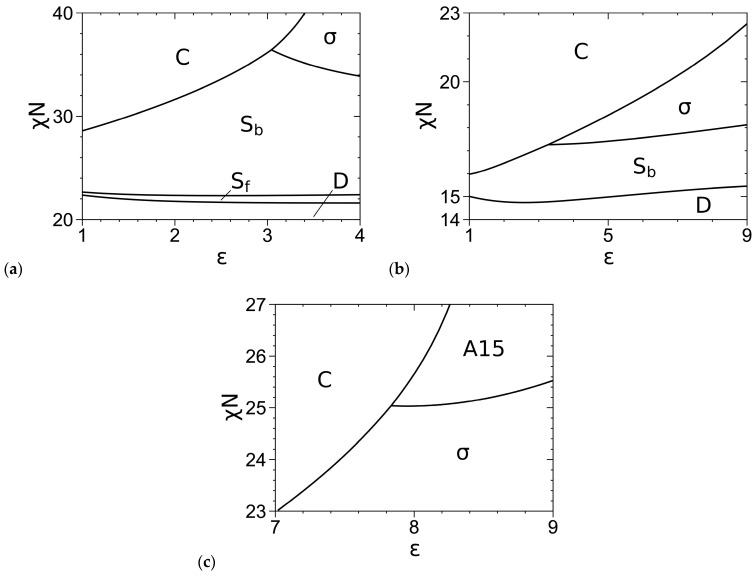
Phase diagrams of the DPDC model at (**a**) *f* = 0.2 and *N* = 10, (**b**) *f* = 0.3 and *N* = 10, and (**c**) *f* = 0.3 and *N* = 20, where “D” denotes the disordered phase. The periodic unit cells of C, C14, σ and all other ordered phases (i.e., S_b_/S_f_/A15/C15/Z) are uniformly discretized into 64^2^, 64 × 64 × 128, 128 × 128 × 64 and 64^3^ grid points, respectively. The phase boundaries are solved to an accuracy of 10^−7^ in *βf_c_* [1]. The triple points are at (*ε*, *χN*) = (3.045, 36.428) in (**a**), (3.290, 17.263) in (**b**), and (7.840, 25.037) in (**c**). *N*/*κ* = 50*π* and $b_{B}/r_{c}=\sqrt{3}/2$ in all the cases. See the main text for more details.

For completeness, we reproduce in Figure 4a,b our SCF phase diagrams of the DPDC model at *N* = 10 reported in Figure 2 of Ref. [1], where *σ* is the only stable FK phase. Figure 4c further shows our SCF phase diagram of this model at *N* = 20 and *f* = 0.3, where A15 emerges as another stable FK phase. Here, the phase boundaries are solved via Ridders’ method [42] by equating, to an accuracy of 10^−7^, *βf_c_* of the two phases having the smallest *βf_c_* at given *ε* and the obtained *χN* [1]. As mentioned in Ref. [1], for the nearly incompressible melts studied here, our previous work for conformationally symmetric A-B shows negligible differences between the SCF phase boundaries determined by equating *βf_c_*, where the two phases have the same density, and those by equating the chain chemical potential and pressure, where the two phases have different densities and the two-phase coexisting region could appear [43].

Comparing Figure 3 and Figure 4, we see that at *f* = 0.2, the SCF phase diagram of the DPDC model at *N* = 10 is qualitatively consistent with that of the “standard” model, but they are qualitatively different at *f* = 0.3 (where A15 is not stable for the DPDC model); the latter and any quantitative differences in the SCF phase diagrams are solely due to the differences between these two models. Changing the “standard” model to the DPDC model expands the stable regions of C and S_b_ into that of σ at both *f*-values and the stable region of C into that of A15 at *f* = 0.3. On the other hand, that A15 becomes stable for the DPDC model at *f* = 0.3 as *N* increases to 20 is consistent with the fact that the DPDC model reduces to the “standard” model [5,6] in the limit of *N→*∞, *b*_P_→0 (at finite *R*, *ε* and $b_{B}/r_{c}$) and *κ→*0.

### 3.3. Curves of βf_c_ and Its Components

**Figure 5:**
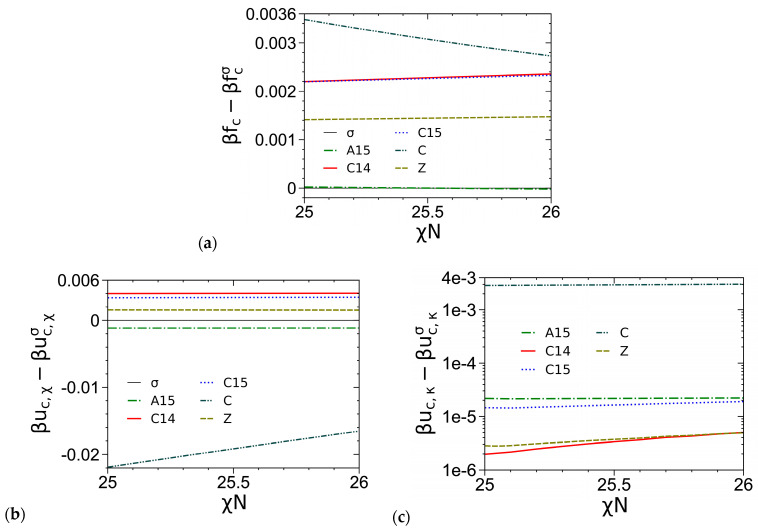

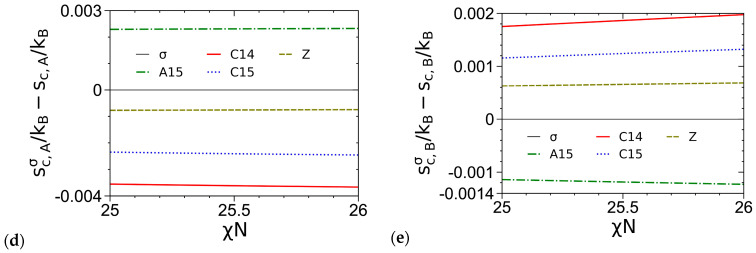
Comparisons of (**a**) the dimensionless (mean-field) Helmholtz free energies per chain *βf_c_*, (**b**) the dimensionless internal energies per chain due to the A-B repulsion *βu_c_*_,*χ*_ and (**c**) those due to the system compressibility *βu_c_*_,*κ*_, and (**d**) the dimensionless entropies per chain of the A block *s_c_*_,A_/*k_B_* and (**e**) those of the B block *s_c_*_,B_/*k_B_*, of various ordered phases obtained from our SCF calculations of the DPDC model, with the σ phase taken as a reference. Due to their too large *βf_c_*, the S_b_ and S_f_ phases are not shown here. Similarly, since for *χN* = 25~26 $s_{c,A}^{\sigma }/k_{B}-s_{c,A}^{C}/k_{B}=0.1322\sim 0.1316$ and $s_{c,B}^{\sigma }/k_{B}-s_{c,B}^{C}/k_{B}=-0.110\sim -0.115$, the C phase is not shown in (**d**,**e**). *N* = 20, *f* = 0.3, *ε* = 9, *N*/*κ* = 50*π* and $b_{B}/r_{c}=\sqrt{3}/2$. See the main text for more details.

As an example, Figure 5a compares the *βf_c_* of various ordered phases obtained from our SCF calculations of the DPDC model at *N* = 20, *f* = 0.3 and *ε* = 9, where we see that A15 becomes stable over σ for *χN* > 25.529 and that their difference in *βf_c_* is about 2 × 10^−5^ or smaller in the figure. Figure 5b–e compare the various components of *βf_c_*, including the dimensionless internal energy per chain *βu_c_*_,*χ*_ due to the A-B repulsion, the dimensionless internal energy per chain *βu_c_*_,*κ*_ due to the system compressibility and the dimensionless entropy per chain *s_c_*_,P_/*k_B_* of the P block, for various ordered phases. Same as the cases of *N* = 10 shown in Ref. [1], we see that the stability of σ (and A15) at intermediate *χN* is due to its delicate balance between *βu_c_* = *βu_c_*_,*χ*_ + *βu_c_*_,*κ*_ and *s_c_*/*k_B_* = *s_c_*_,A_/*k_B_* + *s_c_*_,B_/*k_B_*, and that *βf_c_* = *βu_c_* − *s_c_*/*k_B_* of the FK phases increase in the order of σ/A15, Z, and C14/C15 with their differences being on the order of 10^−3^ or smaller. In addition, our results show that *βu_c_*_,*κ*_ accounts for only 0.3~0.5% of *βu_c_*, thus indicating that our DPDC model at *N*/*κ* = 50*π* is nearly incompressible. 

### 3.4. Effects of N in the DPDC Model

**Figure 6:**
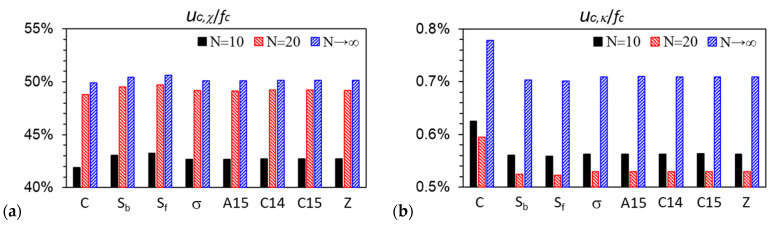

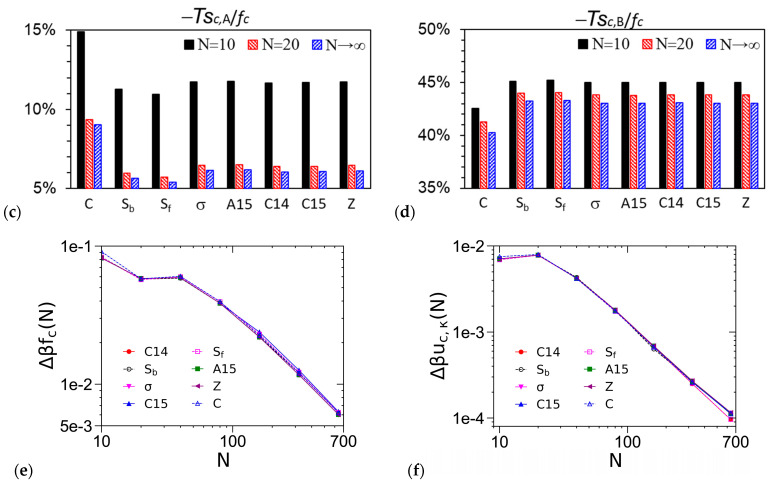
Variations of (**a**) the percentage of the dimensionless internal energy per chain due to the A-B repulsion *βu_c_*_,*χ*_ in the dimensionless Helmholtz free energy per chain *βf_c_*, (**b**) that of the dimensionless internal energy per chain due to the system compressibility *βu_c_*_,*κ*_ in *βf_c_*, (**c**) that of the dimensionless entropy per chain of the A block *s_c_*_,A_/*k_B_* in *βf_c_*, (**d**) that of the dimensionless entropy per chain of the B block *s_c_*_,B_/*k_B_* in *βf_c_*, (**e**) *βf_c_*, and (**f**) *βu_c_*_,*κ*_ vs. the chain length *N*, for various ordered phases obtained from our SCF calculations of the DPDC model, where Δ*βf_c_*(*N*) ≡ |*βf_c_*(*N*) − *βf_c_*(*N→*∞)| and Δ*βu_c_*_,*κ*_(*N*) ≡ |*βu_c_*_,*κ*_(*N*) − *βu_c_*_,*κ*_(*N→*∞)|, respectively, and *N→*∞ corresponds to the Edwards model. *f* = 0.3, *ε* = 9, *χN* = 26, *N*/*κ* = 50*π* and $b_{B}/r_{c}=\sqrt{3}/2$. See the main text for more details.

As mentioned in our recent paper [1], at finite *R*, *ε* and $b_{B}/r_{c}$, as *N→*∞ (thus, *b*_P_→0 and *r_c_→*0), our (compressible) DPDC model becomes the Edwards model [44] (i.e., a compressible diblock copolymer melt, or equivalently a solution in an implicit good solvent, of continuous Gaussian chains with the Dirac *δ*-function non-bonded potential). Figure 6 shows that for all the ordered phases considered in this work, *u_c_*_,*χ*_/*f_c_*, −*Ts_c_*_,A_/*f_c_* and −*Ts_c_*_,B_/*f_c_*, as well as *βu_c_*_,*κ*_ and *βf_c_* for *N* ≳ 30, of the DPDC model approach their corresponding value of the Edwards model monotonically as *N* increases. In addition, as *N* increases from 10 to 20, *u_c_*_,*χ*_/*f_c_* (and −*Ts_c_*_,A_/*f_c_*) exhibits the largest change. Figure 6a shows that *u_c_*_,*χ*_/*f_c_* increases as *N* increases for all the ordered phases considered here; in particular, we note that at *f* = 0.3, *ε* = 9 and *χN* = 26, as shown in the figure, C is the stable phase at *N* = 10, while σ becomes more stable than C at *N* = 20 (note that at both *N*-values, A15 is slightly more stable than σ with a ~10^−5^ lower *βf_c_*). Given the small changes in *βf_c_* of these phases shown in Figure 6e as *N* increases from 10 to 20, our finding that *βu_c_*_,*χ*_ increases as σ becomes more stable than C is consistent with that of Collanton and Dorfman [25], who recently found that *βu_c_*_,*χ*_ increases as the stable phase changes from C to σ while *f* decreases in their SCF calculations of the “standard” model. Finally, Figure 6b again shows that our DPDC model at *N*/*κ* = 50*π* is nearly incompressible.

## 4. Summary

To summarize, we have constructed a self-consistent field (SCF) phase diagram of the dissipative particle dynamics chain (DPDC) model (i.e., compressible melts of discrete Gaussian chains with the DPD non-bonded potential) of conformationally asymmetric diblock copolymers A-B proposed in our recent paper [1] in the *χN*-*ε* plane, where *χN* and *ε* ≡ (*b*_A_/*b*_B_)^2^ ≥ 1 characterize, respectively, the repulsion and conformational asymmetry between A and B blocks with *b*_P_ (P = A, B) being the effective bond length of the P block at the copolymer chain length *N* = 20 and the A-block volume fraction *f* = 0.3. Together with our SCF phase diagrams of this model at *N* = 10 and *f* = 0.2 and 0.3 reported recently [1], we find that σ and A15 are the only stable Frank–Kasper (FK) phases among the five FK (i.e., σ, A15, C14, C15 and Z) phases considered here. The stability of σ and A15 is due to their delicate balance between the energetic and entropic contributions to the dimensionless (mean-field) Helmholtz free energy per chain *βf_c_*. Within our parameter range, *βf_c_* of the FK phases increase in the order of σ/A15, Z, and C14/C15, with their differences being on the order of 10^−3^ or smaller.

We have also constructed SCF phase diagrams of the “standard” model [5,6] (i.e., incompressible melts of continuous Gaussian chains with the Dirac *δ*-function non-bonded potential) in the *χN*-*ε* plane at *f* = 0.2 and 0.3, and compared them with those of the DPDC model. At *f* = 0.2, the SCF phase diagram of the DPDC model at *N* = 10 is qualitatively consistent with that of the “standard” model, but they are qualitatively different at *f* = 0.3 (where A15 is not stable for the DPDC model); any differences in the SCF phase diagrams are solely due to the differences between these two models. On the other hand, A15 becomes stable for the DPDC model at *f* = 0.3 and *N* = 20, consistent with the fact that the DPDC model reduces to the “standard” model in the limit of *N→*∞, *b*_P_→0 (at finite $\sqrt{N/6}b_{B}$, *ε* and $b_{B}/r_{c}$ with *r_c_* denoting the range of the DPD potential) and the generalized Helfand compressibility [36] *κ→*0.

As pointed out in our recent paper [1], SCF theory inherently neglects the effects of system fluctuations/correlations, which are important to the low-molecular-weight diblock copolymer melts forming FK phases in experiments, and direct comparison between SCF and molecular simulation or field-theoretic simulation [30] results based on the same model system, thus without any parameter fitting, is the only way to unambiguously quantify such effects. Since the “standard” model cannot be used in these simulations, this work provides the necessary mean-field reference for unambiguously quantifying the fluctuation/correlation effects with the DPDC model that can be readily used in both molecular and field-theoretic simulations.

## Figures and Tables

**Table 1:** Comparison of various phase boundaries obtained in this work and by Fredrickson and co-workers [3].

	This Work	Ref. [3]
*f* = 0.2 and *χN* = 40		
C/S_b_	*ε* = 1.874	*ε* = 1.882
S_b_/σ	*ε* = 2.667	*ε* = 2.722
*f* = 0.2 and *ε* = 9		
D/S_f_	*χN* = 24.225	*χN* = 24.155
S_f_/S_b_	*χN* = 26.261	*χN* = 26.177
S_b_/σ	*χN* = 30.746	*χN* = 30.483
*f* = 0.3 and *χN* = 40		
C/A15	*ε* = 6.210	*ε* = 6.249
*f* = 0.3 and *ε* = 9		
S_b_/σ	*χN* = 17.303	*χN* = 17.257
σ/A15	*χN* = 25.629	*χN* = 25.629

## Data Availability

The data presented in this study are available upon reasonable request from the corresponding author.
